# The pediatric nasal microbiome and its role in chronic ENT disorders: a narrative review

**DOI:** 10.3389/fimmu.2025.1699707

**Published:** 2026-01-14

**Authors:** Adriana Elena Sîrbu, Gratiela Gradisteanu Pircalabioru, Diana Maria Deaconu, Octavian Andronic, Dan Cristian Gheorghe

**Affiliations:** 1 Faculty of Medicine, “Carol Davila” University of Medicine and Pharmacy, Bucharest, Romania; 2 ENT Department, “Marie Curie” Children Hospital, Bucharest, Romania; 3 Department of Botany and Microbiology, Faculty of Biology, University of Bucharest, Bucharest, Romania; 4 Research Institute of University of Bucharest, Bucharest, Romania; 5 eBio-Hub Centre of Excellence in Bioengineering, National University of Science and Technology Politehnica Bucharest, Bucharest, Romania; 6 Innovation and eHealth Center, Carol Davila University of Medicine and Pharmacy Bucharest, Bucharest, Romania

**Keywords:** adenoid and tonsillar hypertrophy, allergic rhinitis, dysbiosis, nasal microbiome, otitis media, pediatric

## Abstract

The human microbiome is increasingly recognized as a key factor in immune development and disease susceptibility, especially in early life. Nasal microbiome has emerged as a critical element in upper airway health, yet its role in pediatric otorhinolaryngological conditions remains underexplored. This narrative review synthesizes current evidence on the microbial nasopharyngeal patterns in healthy children compared with children suffering from chronic ENT conditions such as otitis media, allergic rhinitis, chronic rhinosinusitis, adenoid and tonsillar hypertrophy associated with obstructive sleep apnea. A structured search of Web of Science, PubMed, Google Scholar and CrossRef databases was conducted for peer-reviewed articles published in the past ten years. Nasal microbiota of healthy children was proved to be dominated by commensal protective taxa such as *Dolosigranulum* and *Corynebacterium* which contribute to mucosal immune stability. In contrast, patients with chronic ENT pathologies exhibited reduced diversity and increased prevalence of potential pathogens microbial species such as *Haemophilus*, *Streptococcus* and *Staphylococcus.* Several extrinsic factors appear to play an important role in modulating the nasal microbiota such as environmental exposure, delivery mode, feeding practices and antibiotic treatment. Growing evidence supports the predictive and modulatory potential of the nasal microbiome, however methodological variability, limited pediatric-specific studies and unclear causal relationships remain challenging components. This review highlights key microbial patterns, outlines the limitations of current research and suggests future directions for clinical integration of nasal microbiome analysis in pediatric ENT standard of care as it may hold promising utilisation of biomarkers for disease risk stratification and targeted therapeutic or preventative interventions in early life.

## 1 Introduction

Many areas of medicine have been transformed by understanding the impact of human microbiome on health, otorhinolaryngology being no exception (1). A key site of microbial interactions is the upper respiratory tract, where the immune system and mucosal barriers are still developing in children (2). The nasopharyngeal microbiota in early life not only supports local immune homeostasis but may also shape the risk of recurrent infections or chronic inflammation (3).

Although the interest for studying human microbiome has increased among the years, research into the nasopharyngeal microbiome, especially in children, is still emerging especially in pediatric populations (2, 3). In maintaining the mucosal health, several bacterial genera, such as *Dolosigranulum pigrum (*D. *pigrum)* and *Corynebacterium accolens (*C. *accolens)*, appear to play a protective role (4, 5), while others like *Haemophilus influenzae (*H. *influenzae), Moraxella catarrhalis (*M. *catarrhalis)*, and *Streptococcus pneumoniae* (S. p*neumoniae*) are frequently associated with respiratory infections (2, 6).

Microbial patterns raise relevant clinical questions regarding their potential as a predictive biomarker for ENT diseases and their usage for prevention or targeted therapies (7, 8). External factors such as antibiotic exposure, delivery mode, environmental pollution or passive smoke can influence microbial colonization especially in early life, possibly impacting the development of diseases (3, 7). Despite these advances, the research field still lacks consensus on standard sampling methods, microbial thresholds or consistent links between microbiota patterns and specific disorders in children (1, 2).

This narrative review aims to synthesize current evidence on the pediatric nasopharyngeal microbiome and its associations with common otorhinolaryngological conditions, including otitis media, chronic adenoiditis, allergic rhinitis and recurrent sinusitis. In addition to characterizing dominant microbial taxa, this review also highlights knowledge gaps, methodological challenges and the clinical potential of microbiome-based approaches in pediatric ENT practice.

## 2 Literature search strategy

This narrative review is based on a structured and exploratory literature search aimed at identifying peer-reviewed studies relevant to the pediatric nasal microbiome and its association with chronic otorhinolaryngological pathologies. The research included databases such as PubMed, Web of Science, Google Scholar and CrossRef. Although the primary literature search targeted publications from 2014 to 2024, earlier studies were included due to their foundational contribution and continued relevance to the field. Key words used for search included combinations of “nasal microbiome”, “nasopharyngeal microbiota”, “children”, “otitis media”, “adenoid hypertrophy”, “allergic rhinitis”, “chronic rhinosinusitis” and “pediatric sleep apnea”.

In addition, references were identified through manual screening of bibliographies from relevant papers, targeted journal browsing and recommendations retrieved through academic tools. Only studies published in English, involving human pediatric populations and deemed scientifically rigorous were included. Inclusion criteria comprised peer-reviewed original research articles or high-quality reviews involving human pediatric populations (from birth to 18 years) that addressed the nasal or nasopharyngeal microbiome—including bacterial, viral, or fungal components—in health or ENT disease contexts and were published in English. Studies conducted exclusively in adults or animal models, case reports, conference abstracts, editorials, and non–peer-reviewed literature were excluded, as were studies focusing solely on the oral or gut microbiome without relevance to the nasal cavity or those lacking microbiome-specific data or methodological clarity. As this work represents a narrative review, no formal risk-of-bias assessment or quantitative meta-analysis was performed; however, priority was given to well-designed cohort studies, longitudinal investigations, and studies employing high-resolution sequencing or multi-omics approaches to ensure thematic relevance, methodological robustness, and clinical applicability.

## 3 The nasal microbiome in healthy children

### 3.1 Colonization in early life

The establishment of a healthy nasal microbiome is a dynamic, multi-step process that begins at birth and continues throughout early childhood, shaped by a complex interplay of host-related, environmental, and microbial factors. Rather than forming randomly, nasal microbial communities assemble in a predictable yet plastic manner, reflecting both early-life exposures and ongoing ecological pressures.

The nasal microbiome development starts from birth and suffers significant changes in the first two to three years of life. The birth method has an impact on infant microbiota as vaginal births result in microbiota similarly to vaginal flora, whereas caesarean sections lead to lower bacterial diversity and a high prevalence of skin-associated taxa (9, 10). These early differences may influence microbial diversity trajectories and immune priming during critical developmental windows.

Another important factor is feeding in early life when it comes to microbial colonization. Breastfeeding supports the development of protective commensals such as D. *pigrum* and C. *accolens* and has been associated with reduced susceptibility to upper tract infections, while infants fed with infant formula may display an alternative microbial trajectory (11, 12).

Other parameters must be taken into consideration including maternal microbiota, administration of antibiotic in the perinatal period and caregiver contact, which influence microbial stabilization (13, 14). Long-term respiratory health is thus impacted by the dynamics of microbial colonization in early life.

**Figure 1:**
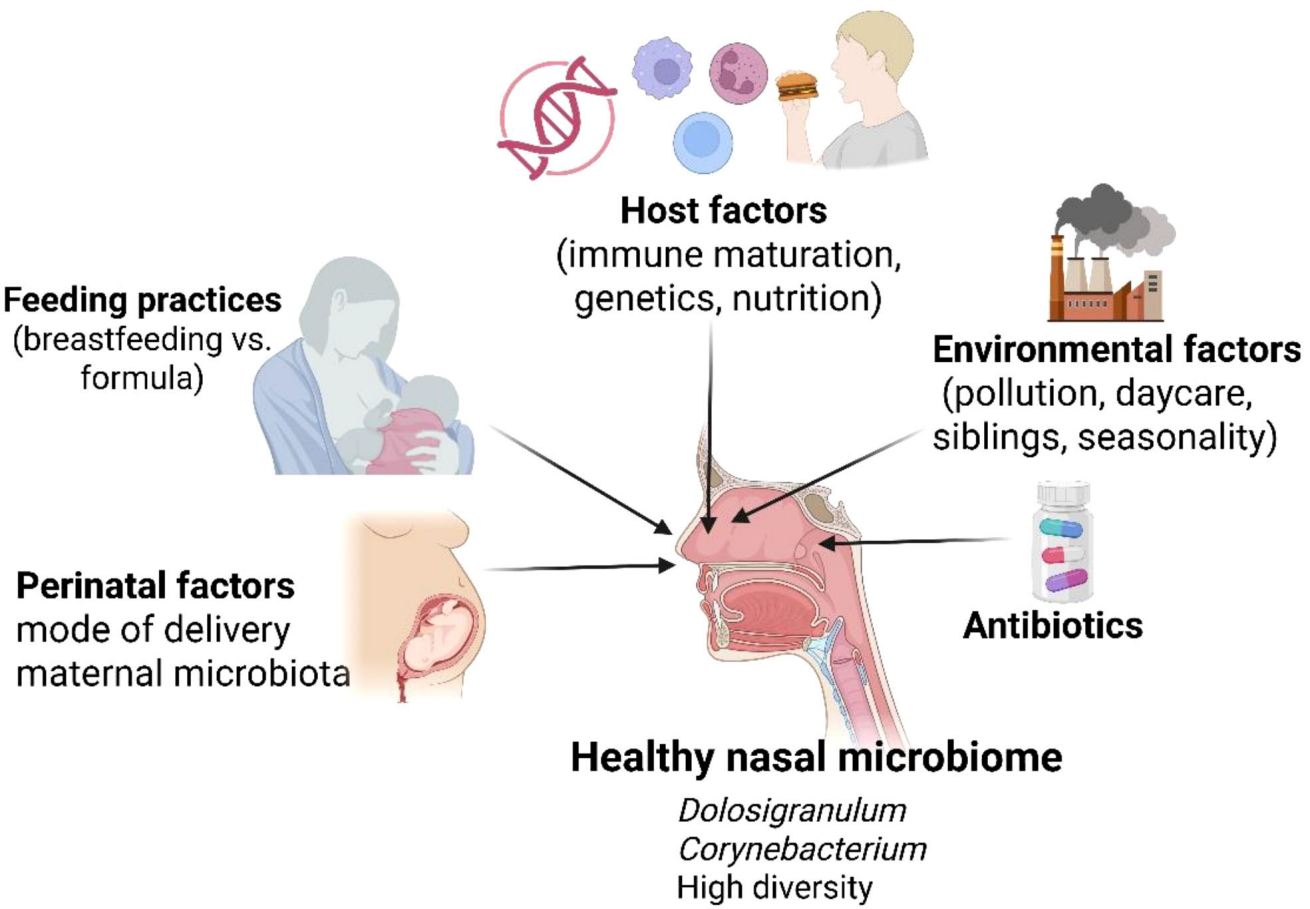
Factors influencing the establishment of a healthy nasal microbiome in early life. The pediatric nasal microbiome is shaped by perinatal, environmental, microbial, and host-related factors, including mode of delivery, feeding practices, antibiotic exposure, environmental conditions, and immune maturation. These determinants collectively influence microbial diversity, commensal dominance, and ecosystem resilience, directing the nasal microbiome toward either homeostasis or dysbiosis and subsequent ENT disease risk.

Environmental factors strongly influence nasal microbiome maturation (**Figure 1**). Exposure to siblings, daycare attendance, and household crowding increases microbial exchange and diversity, while urban pollution and passive tobacco smoke exposure have been associated with shifts toward pro-inflammatory microbial profiles (12–14). Seasonality also plays a role, with colder months favoring increased carriage of respiratory pathogens, likely due to viral co-infections and altered mucosal immunity. Host-related factors, including genetic background, immune system maturation, and nutritional status, further shape microbial composition (13). The developing mucosal immune system actively selects for tolerated commensals while restricting pathogenic expansion through antimicrobial peptides, secretory IgA, and epithelial barrier mechanisms. This bidirectional host–microbe interaction contributes to the stabilization of a health-associated microbial ecosystem.

Collectively, these factors determine whether the nasal microbiome evolves toward a commensal-dominated, resilient state or remains vulnerable to dysbiosis and disease. Understanding these determinants is essential for identifying critical windows for preventive interventions and microbiome-informed strategies in pediatric ENT care.

### 3.2 Core microbial taxa and health-associated profiles

Beyond the mere absence of pathogens, a healthy pediatric nasal microbiome represents a structured and functionally active microbial ecosystem that contributes to mucosal homeostasis, immune education, and protection against disease (1). In early life, the nasopharyngeal niche is typically dominated by a limited number of commensal taxa whose presence and relative abundance are increasingly recognized as hallmarks of respiratory health.

The nasal microbiome in healthy children is typically composed of a limited set of dominant genera, including D. *pigrum*, C. *accolens*, M. *catarrhalis* and *Staphylococcus epidermidis* (S. *epidermidis*) (11, 15, 16). Among these, *Dolosigranulum pigrum* and *Corynebacterium accolens* consistently emerge as core members of health-associated nasal microbial profiles in infants and young children Multiple cohort studies have demonstrated that the co-dominance of *D. pigrum* and *Corynebacterium* species correlates with reduced susceptibility to upper respiratory tract infections, otitis media, and chronic inflammatory ENT conditions. This microbial configuration is considered a marker of ecosystem stability and resilience in the developing upper airway (4, 5).

Functionally, these commensal bacteria exert protective effects through several complementary mechanisms. *Corynebacterium accolens* is known to metabolize host-derived skin and mucosal lipids, releasing free fatty acids with potent antipneumococcal and anti-*Staphylococcus aureus* activity. Through this metabolic activity, *Corynebacterium* species actively contribute to competitive exclusion, limiting pathogen colonization without triggering inflammation. In parallel, *D. pigrum* has been associated with reinforcement of epithelial barrier integrity and modulation of local immune responses, including reduced expression of pro-inflammatory cytokines and promotion of immune tolerance at the mucosal surface. Although the precise molecular pathways remain under investigation, accumulating evidence suggests that *D. pigrum* may indirectly support epithelial maturation and mucin regulation, thereby strengthening the first line of defense against airborne pathogens (15).

Another important constituent of the healthy nasal microbiome is *Staphylococcus epidermidis*. While traditionally regarded as a skin commensal, *S. epidermidis* plays a relevant role in the nasal cavity by inhibiting colonization by more virulent staphylococcal species, particularly *S. aureus* (17). This antagonistic effect is mediated through both competition for ecological niches and the production of antimicrobial peptides, further contributing to microbial balance and protection against infection.

Importantly, not all taxa detected in healthy children are strictly beneficial under all circumstances. Certain organisms, such as *Moraxella catarrhalis*, exemplify the concept of pathobionts—microbes that can exist as harmless commensals during early colonization but possess the capacity to promote inflammation or disease when ecological balance is disrupted. In the context of a stable and diverse microbiome dominated by protective taxa, *M. catarrhalis* may persist without adverse effects (6, 15). However, factors such as viral infections, antibiotic intake, or loss of commensal competitors can shift this balance, allowing pathobionts to expand and contribute to mucosal inflammation, biofilm formation, and disease progression.

Respiratory tract pathogens are thought to be inhibited by *Corynebacterium* species using the production of free fatty acids and other metabolic by-products (18). The function of mucosal barrier may be reinforced by D. *pigrum* while positively interacting with host immunity system, though the mechanism of this process is still researched (19).

In general, an increased relative abundance of D. *pigrum* and C. *accolens*, alongside a reduced prevalence of potential pathogenic taxa, has been linked to improved mucosal immune homeostasis and a decreased susceptibility to otorhinolaryngologic infections during early childhood (4, 11).

Collectively, these observations highlight that the healthy pediatric nasal microbiome is not a passive microbial assemblage but an active regulator of upper airway physiology. Through metabolic activity, immune modulation, and inter-microbial interactions, commensal taxa shape epithelial barrier function and immune tolerance, while restraining opportunistic pathogens. Disruption of this finely tuned ecosystem—rather than the presence of a single pathogen—appears to be a critical step in the pathogenesis of chronic ENT disorders in children.

### 3.3 Factors influencing microbiome stability and diversity

Nasal microbiome is affected by several external and host-related factors especially in children. Exposure to antibiotics, particularly in the first year of life, has been associated with decreased microbial diversity and delayed return to equilibrium (12). Urban living conditions, pollution and passive tobacco smoke exposure also contribute to microbial shifts favouring pathogenic taxa (15, 20).

The composition of microbiota appears to be influenced by seasonality, with colder months associated with increased colonization by respiratory pathogens (3) Socioeconomic factors, including household crowding and daycare attending, have been shown to affect microbial diversity (21). Furthermore, through systemic immunological effects, dietary patterns and nutritional status may indirectly modulate the nasal microbiome (22).

### 3.4 Immunological relevance and host-microbiota interaction

The nasal and nasopharyngeal mucosa represent critical immunological interfaces where host defenses continuously interact with resident microbial communities (23). Far from being passive barriers, these mucosal surfaces actively shape microbial community structure through epithelial integrity, innate immune signaling, and adaptive immune responses (24). In turn, the composition and functional activity of the nasal microbiome profoundly influence mucosal immune tone, contributing to either immune homeostasis or chronic inflammation.

In healthy children, a commensal-dominated nasal microbiome supports mucosal immune equilibrium. Epithelial cells, together with resident immune cells, recognize commensal microbes through pattern recognition receptors such as Toll-like receptors, leading to controlled immune activation without excessive inflammation. Health-associated taxa, including *D.pigrum* and *C. accolens*, have been linked to reduced pro-inflammatory cytokine expression, enhanced production of antimicrobial peptides, and promotion of regulatory immune pathways (25). Local immune responses are being highly influenced by nasopharyngeal microbiome’s development during early childhood. D. *pigrum* and C. *accolens* are commensal bacteria involved in epithelial barrier maturation, mucin regulation and modulation of local cytokine profiles (4, 26). Their presence has been associated with the induction of regulatory T cells and suppression of pro-inflammatory reactions (27). These interactions support epithelial barrier maturation, mucin regulation, and effective mucociliary clearance, collectively reinforcing the first line of defense against inhaled pathogens.

Disruption of this balanced ecosystem—whether through infection, antibiotic exposure, or environmental stressors—can lead to microbiome-driven immune dysregulation. Dysbiosis is the process defined by increased pathogenic taxa and reduced commensal abundance. It is associated with mucosal inflammation and higher susceptibility to infection (6, 21). The dynamic interactions between microbial colonies and host’s immunity responses are playing an important role in microbial species selection (27).

**Figure 2:**
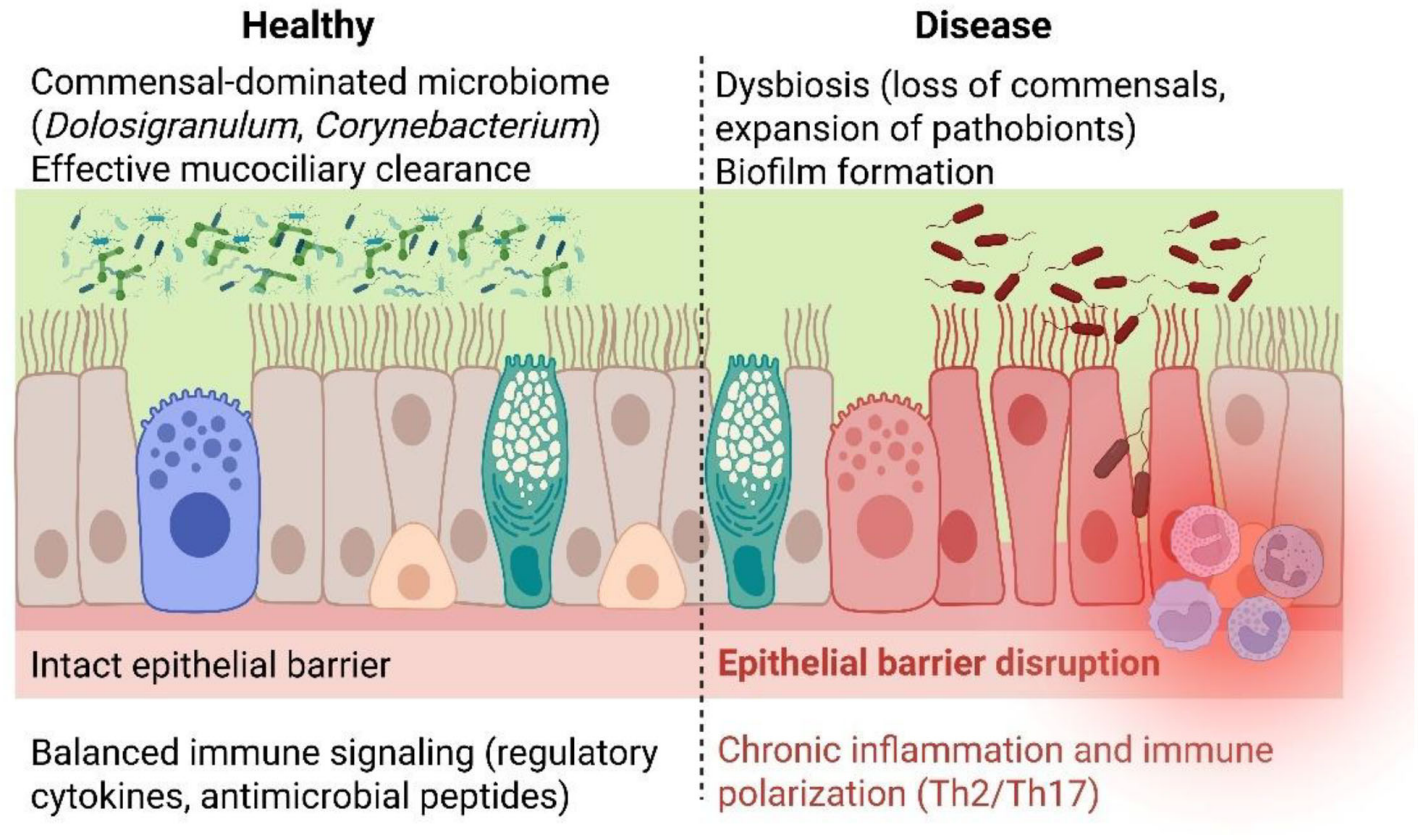
Interactions between the nasal microbiome and mucosal immunity in pediatric ENT health and disease. In health, commensal-dominated microbial communities support epithelial barrier integrity, immune tolerance, and mucociliary clearance. Disruption of this balance leads to dysbiosis, epithelial damage, immune polarization, and biofilm formation, creating self-reinforcing inflammatory loops that underlie chronic ENT disorders such as otitis media, allergic rhinitis, adenoid hypertrophy, and chronic rhinosinusitis.

Loss of protective commensals and reduced microbial diversity are frequently accompanied by increased abundance of inflammation-tolerant or pro-inflammatory taxa such as *Haemophilus influenzae, Moraxella catarrhalis, Streptococcus pneumoniae*, and *Staphylococcus aureus* (27, 28). These organisms can actively modulate host immunity by inducing epithelial cytokine release, skewing local immune responses toward Th1, Th2, or Th17 polarization, and impairing regulatory pathways. Such immune shifts are particularly relevant in allergic rhinitis, where microbiome alterations may amplify type 2 inflammation, and in chronic rhinosinusitis, where persistent immune activation sustains mucosal edema and tissue remodeling (**Figure 2**).

A central mechanistic link between microbiome alterations and chronic ENT disease is epithelial barrier dysfunction. Inflammatory mediators released during dysbiosis disrupt tight junction integrity, increase epithelial permeability, and impair mucociliary clearance. This compromised barrier facilitates deeper microbial penetration and prolonged antigen exposure, further perpetuating inflammation. In children, whose mucosal barriers are still developing, this process may be particularly impactful, predisposing to recurrent infections and chronic disease.

Another key pathogenic mechanism is biofilm formation (29). Under dysbiotic conditions, pathobionts can organize into biofilms on the nasal epithelium, adenoids, tonsils, or sinus mucosa (30). Biofilms provide physical protection against host immune defenses and antimicrobial treatments, allowing bacteria to persist in a low-grade inflammatory state (29–31). Biofilm-associated communities exhibit altered gene expression, enhanced resistance, and cooperative metabolic interactions, all of which contribute to disease chronicity in conditions such as otitis media with effusion, adenoid hypertrophy, and chronic rhinosinusitis (31).

Importantly, mucosal inflammation and microbial dysbiosis engage in self-reinforcing feedback loops. Inflammation alters the local microenvironment—through changes in oxygen availability, nutrient composition, and antimicrobial peptide expression—selectively favoring microbes adapted to inflammatory conditions (32). These microbial shifts further stimulate immune activation, creating a cycle that stabilizes disease-associated microbial communities and prevents restoration of a healthy, commensal-dominated state.

## 4 The nasal microbiome in ENT pathology in children

Several research studies have demonstrated the role of nasopharyngeal microbiota in various pediatric ENT disorders. Dysbiosis is associated with chronic inflammation, pathogen overgrowth and impaired mucosal immunity as previously shown.

### 4.1 Otitis media

Otitis media (OM) is one of the most common infectious diseases in paediatrics and a leading cause of antibiotic prescriptions and surgical interventions. Predisposition to episodes of OM and its clinical course is currently considered to be linked to nasopharyngeal microbiome, as several new evidence supports this hypothesis (33, 34). Otitis media is most strongly associated with alterations of the nasopharyngeal microbiota, rather than the anterior nasal or oral microbiota. The nasopharynx serves as the primary ecological reservoir for otopathogens such as *Haemophilus influenzae*, *Moraxella catarrhalis*, and *Streptococcus pneumoniae*, which can ascend via the Eustachian tube to the middle ear. In contrast, the anterior nasal cavity is more frequently dominated by commensal taxa such as *Dolosigranulum* and *Corynebacterium*, while the oropharynx and oral cavity harbor more complex and anaerobe-rich communities (33–37).

In healthy children, microbiota of nasopharynx is generally dominated by commensals species of *Corynebacterium* and *Dolosigranulum*, which are associated with mucosal protection and reduced incidence of OM (35). At the opposite end, children with recurrent OM or chronic otitis media with effusion (OME) express microbial profiles marked by higher levels of pathogens such as *H. influenzae, M*. *catarrhalis* and S. *pneumoniae* (36, 37).

**Figure 3:**
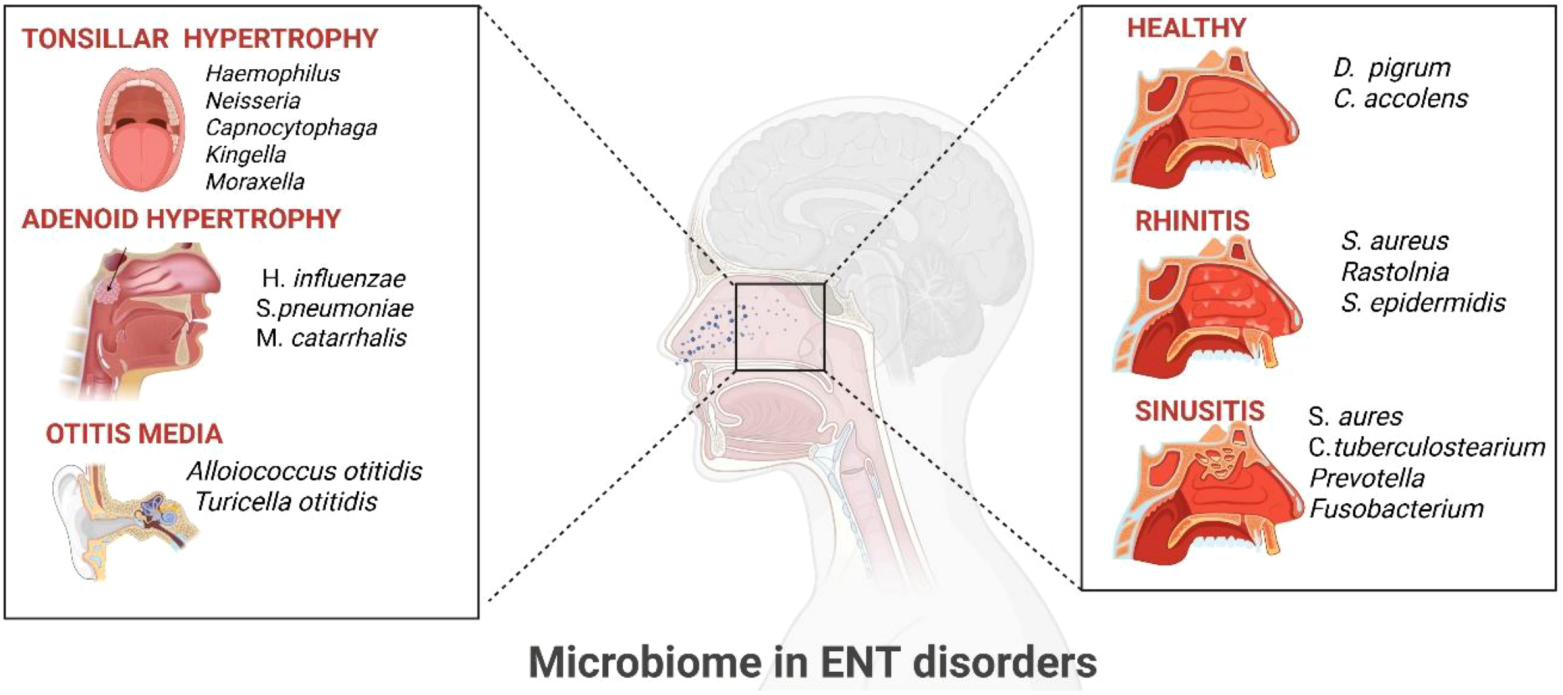
Microbiome in ENT disorders. Distinct microbial patterns are associated with ENT conditions: *Haemophilus, Neisseria*, and *Moraxella* in tonsillar/adenoid hypertrophy, *Alloiococcus* and *Turicella* in otitis media, while the nasal cavity shows *Dolosigranulum* and Corynebacterium in health, but *Staphylococcus, Ralstonia, Prevotell*a, and *Fusobacterium* in rhinitis and sinusitis.

Children with OME often present with lower microbial diversity and higher abundance of otopathogens using 16S rRNA sequencing as research studies have proven (38) (**Figure 3**). *Alloiococcus otitidis* (A. *otitidis*), previously considered a contaminant or benign colonizer, has emerged as a possible factor to middle ear inflammation, particularly in the presence of biofilms (39). Similarly, *Turicella otitidis* (T. *otitidis*) has been researched in some cohorts, although its clinical significance remains unclear (40).

These microbial shifts have been detected using 16S rRNA sequencing and shotgun metagenomics, revealing altered networks between nasopharynx and middle ear. Several studies suggest that early microbiome patterns may predict the risk of future OM, underlining the potential for microbial biomarkers to develop individualized prevention strategies (1, 32).

Several host and environmental factors known to influence early microbiome development, such as birth mode, feeding practices and antibiotic exposure, have been linked to OM susceptibility, as detailed above.

Microbiota-targeted approaches including probiotics, bacterial interference and narrow-spectrum antibiotics have been proposed to preserve commensal taxa and reduce the risk of recurrent infections, though clinical evidence remains limited (41).

### 4.2 Allergic rhinitis

Allergic rhinitis (AR) is a prevalent chronic inflammatory condition in children, characterized by nasal congestion, rhinorrhea, sneezing and itching. New evidence suggests that changes in the nasal microbiome may play a role in the pathogenesis and severity of AR (42, 43).

Microbial patterns have been recently studied between children with AR and healthy controls and indicated significant differences. Specifically, there is a marked increase in the abundance of S. *aureus* and S. *epidermidis* in the nasal cavities of AR patients, while beneficial commensals are reduced (42). The inflammatory responses may be stimulated by a shift towards dysbiosis, process observed in patients with AR (44).

In addition, the symptom severity has been shown to correlate with microbial composition of the nasal cavities. Higher levels of species of *Ralstonia* have been associated with more severe nasal symptoms, suggesting a potential role in exacerbating mucosal inflammation (42).

Understanding the nasal microbiome’s role in pediatric AR may pave the way for novel diagnostic or therapeutic strategies. Modulation of microbial colonies could become a future target to improve quality of life in children with AR (43).

### 4.3 Adenoid and tonsillar hypertrophy in paediatric obstructive sleep apnea

Adenoid hypertrophy (AH) and tonsillar hypertrophy (TH) are common causes of upper airway obstruction in children and represent key anatomical substrates for pediatric sleep apnea (OSA) (45). Classically it was though that these conditions are only playing a mechanical role in OSA by obstructing the nasopharynx, however recent studies using sequencing technologies have revealed that there is an important involvement of complex interactions between microbiome and local immune system (46).

Children with AH harbour distinct nasal microbiota compared to healthy controls, with reduced alpha diversity and increased abundance of potential pathobionts as H. *influenzae*, S. *pneumoniae* and M. *catarrhalis*, as recent studies have shown (47). This raises the question if targeting microbial composition early in life could modulate disease progression or severity.

Current management of OSA caused by AH or TH focuses on surgical removal of hypertrophic tissue. Future research of microbial patterns may help ENT surgeons on decision-making between surgery treatment of children with OSA or non-surgical interventions based on a scale of risks (45, 48).

Recent work comparing surface and core tonsillar microbiota in chronic tonsillitis (CT) and tonsillar hypertrophy (TH) patients using 16S rRNA sequencing revealed distinct microbial signatures*. Haemophilus, Streptococcus, Neisseria, Capnocytophaga, Kingella, Moraxel*la, and *Lachnospiraceae* were enriched in TH, while Dialister, Parvimonas, Aggregatibacter, and Atopobium were associated with CT. Network analysis highlighted Dialister, Parvimonas, and Neisseria as key taxa, and a unique microbiota type dominated by Haemophilus and Neisseria was exclusive to TH. These findings suggest specific bacterial genera may serve as biomarkers to discriminate CT from TH and highlight potential microbiota–immune interactions in tonsillar disease (49).

### 4.4 Chronic rhinosinusitis

Chronic rhinosinusitis (CRS) in children is a persistent inflammatory disease of the paranasal sinuses, often leading to nasal obstruction, purulent rhinorrhea, facial pressure and cough lasting more than 12 weeks. There are multiple factors which contribute to the onset of CRS, but recent studies suggest that dysbiosis of the nasal microbiome plays an important role to its pathogenesis and clinical heterogeneity (50, 51).

Children with CRS often present disproportion of microbial diversity with increased expression of pro-inflammatory of biofilm-associated taxa in the nasal cavity, such as S. *aures*, *Corynebacterium tuberculostearicum* (*C. tuberculostearium*), *Prevotella* and *Fusobacterium* (50, 52). These alterations may facilitate persistent mucosal inflammation, impair mucociliary clearance and promote the formation of chronic biofilms (53).

Conversely, in CRS patients there are relatively few cases of protective commensals such as *Dolosigranulum* and *C*. *accolens*, which occur naturally in healthy nasal microbiota (54). This imbalance may facilitate the domination of pathogenic bacteria and contribute to recurrent or treatment-resistant disease.

Furthermore, studies using16S rRNA sequencing have shown that microbial patterns can vary significantly between CRS phenotypes, including those with and without nasal polyps, although data in pediatric populations remain limited (55).

Currently, the standard treatment for CRS in children is based on antibiotics therapy or surgery leading to dysbiosis and antibiotic-resistant bacteria. Future research is necessary to include microbiota-targeted therapies designed to restore nasal microbial equilibrium.

## 5 The nasal virome, mycobiome and antimicrobial resistance in pediatric ENT disorders

In addition to bacterial communities, the healthy pediatric nasal ecosystem comprises a diverse virome and mycobiome that contribute to microbial regulation and immune maturation, even in the absence of overt disease. Although historically underexplored, growing evidence indicates that these non-bacterial components are integral to maintaining ecological balance and mucosal homeostasis in the upper airway.The nasal virome in healthy children is dominated not only by transient respiratory viruses but also by a substantial population of bacteriophages, which play a critical regulatory role within the microbial community (56). Bacteriophages contribute to bacterial population control through predation, limiting the overgrowth of potentially pathogenic taxa and promoting microbial diversity. By selectively targeting dominant or opportunistic bacteria, phages help stabilize commensal-dominated ecosystems and may indirectly support the persistence of health-associated taxa such as *Dolosigranulum* and *Corynebacterium*. In addition, phage-mediated horizontal gene transfer can influence bacterial adaptability and resilience, shaping microbial succession during early-life colonization (57). Importantly, in healthy mucosa, phage activity is thought to occur without inducing inflammation, suggesting a role in immune-tolerant microbial surveillance rather than pathogenic disruption.

Beyond microbial regulation, early-life viral exposures may also contribute to immune conditioning. Low-level or asymptomatic viral encounters in infancy are thought to participate in the maturation of innate antiviral responses and mucosal immune competence (58). This process may enhance epithelial responsiveness and immune readiness while avoiding excessive inflammation, thereby promoting tolerance to commensal microbes and environmental antigens later in life. Respiratory viruses such as rhinovirus, respiratory syncytial virus (RSV), and influenza frequently interact with bacterial colonizers to shape mucosal immunity and disease risk (32, 59). For example, viral infections can disrupt microbial homeostasis, facilitating secondary bacterial colonization and recurrent otitis media, while bacteriophages may regulate bacterial abundance and horizontal gene transfer, thereby influencing microbial resilience. Similarly, the fungal communities of the nasal cavity are underexplored in children. Fungi such as *Malassezia, Candida*, and *Aspergillus* have been detected in the nasal passages and paranasal sinuses, with evidence suggesting that fungal dysbiosis contributes to chronic rhinosinusitis and may amplify allergic inflammation (60).

### 5.1 The nasal virome

The pediatric nasopharynx harbors a diverse community of commensal and pathogenic viruses, including bacteriophages that modulate bacterial dynamics. Viral–bacterial interactions appear to shape mucosal homeostasis and disease risk. For instance, colonization by *Haemophilus influenzae* or *Moraxella catarrhalis* often precedes viral infections such as respiratory syncytial virus (RSV) or rhinovirus, suggesting that microbial imbalance may predispose to viral persistence and secondary bacterial infection. Conversely, recurrent viral infections may drive bacterial dysbiosis and chronic inflammation of the upper airways (61). Longitudinal birth cohorts have shown that early-life viral colonization is associated with subsequent susceptibility to wheezing disorders and asthma, raising the possibility of similar links with recurrent otitis media and chronic rhinosinusitis in children (62).

### 5.2 The nasal mycobiome

The fungal component of the nasal microbiome remains largely overlooked in pediatrics. Nevertheless, fungi such as *Candida, Malassezia, Aspergillus* and *Alternaria* have been detected in the nasal cavity and paranasal sinuses. In health, these fungi coexist with bacterial communities in a state of controlled equilibrium, shaped by interkingdom interactions and host immune surveillance. Their role in ENT disorders is not yet fully understood, but fungal dysbiosis may contribute to chronic rhinosinusitis with or without nasal polyps, particularly in atopic children. Fungal antigens can act as potent immunostimulants, promoting Th2-biased responses and enhancing allergic inflammation. The integration of fungal community profiling with bacterial and viral analysis may help clarify their synergistic roles in mucosal immunity and disease progression.

Indeed, fungal–bacterial interactions represent an important but underexplored factor in upper airway disease (63). These microorganisms co-exist within the sinonasal mucosa, where interkingdom interactions can modulate growth, virulence, and host immunity. For instance, *Staphylococcus aureus* can enhance fungal tissue invasion, while *Pseudomonas aeruginosa* exerts antifungal effects through metabolites such as pyocyanin and phenazine (64). Conversely, *Aspergillus fumigatus* can suppress *P. aeruginosa* growth, highlighting reciprocal antagonism (65). Synergistic interactions, such as between *Malassezia sympodialis* and *S. aureus*, exacerbate type 2 and type 17 immune responses, contributing to chronic sinonasal inflammation (66). Antibiotic-mediated reductions in bacterial burden can shift fungal composition, influencing mucosal inflammation and potentially aggravating chronic rhinosinusitis (CRS). These findings underscore that fungal–bacterial interactions are species-specific and tightly linked to host immune modulation, suggesting that targeting such microbial networks may provide new therapeutic strategies in CRS (63).

Taken together, the inclusion of the virome and mycobiome underscores that nasal health is maintained by a multi-kingdom microbial network, rather than bacteria alone. These components collectively contribute to microbial regulation, immune education, and barrier integrity in early life. Their integration into the concept of a healthy nasal microbiome provides a more comprehensive and biologically grounded framework for understanding resilience against upper airway disease and the consequences of dysbiosis in pediatric ENT disorders.

### 5.3 Antimicrobial resistance

The nasal cavity represents a major ecological reservoir for antimicrobial resistance (AMR). In particular, colonization by *Staphylococcus aureus*, including methicillin-resistant strains (MRSA), is well documented in children (67). Carriage is influenced by both host factors (immune status, prior antibiotic exposure) and ecological balance within the microbiome (68). Dysbiosis that reduces protective commensals such as *D. pigrum* and *C. accolens* may facilitate pathogen overgrowth and horizontal gene transfer, amplifying AMR dissemination. This is particularly concerning in the context of frequent antibiotic use for recurrent otitis media or rhinosinusitis. Characterizing the nasal resistome — the collection of resistance genes — could provide important insights into the spread of AMR in pediatric populations and inform antibiotic stewardship strategies (69).

### 5.4 Microbial community dynamics following pathogenic invasion

Pathogenic invasion of the upper airway does not occur in isolation but rather triggers profound and often sustained alterations in the structure and function of the nasal microbial ecosystem (70). Increasing evidence indicates that viral and bacterial infections act as ecological perturbations, disrupting commensal-dominated communities and initiating shifts toward dysbiosis that may persist beyond the acute infectious episode.

One of the earliest and most consistent changes observed following pathogenic invasion is the loss of commensal dominance and a reduction in alpha diversity (70). Acute viral infections, such as those caused by rhinovirus, respiratory syncytial virus (RSV), or influenza, can directly damage the epithelial barrier, alter mucosal secretions, and modify local oxygen tension and nutrient availability (71). These changes create a permissive environment for opportunistic taxa while disadvantaging health-associated commensals such as *Dolosigranulum pigrum* and *Corynebacterium accolens*. Longitudinal studies in infants and young children have shown that viral respiratory infections are frequently followed by a decline in these protective taxa, accompanied by delayed recovery of microbial diversity (72, 73).

Concomitantly, pathobionts such as *Moraxella catarrhalis, Haemophilus influenzae*, *Streptococcus pneumoniae*, and *Staphylococcus aureus* often expand in relative abundance. While these organisms may be present at low levels in healthy individuals, pathogenic invasion and immune-mediated environmental changes can shift their behavior toward virulence (71–73). This transition is frequently associated with increased expression of adhesins, toxins, and immune-evasive mechanisms, facilitating persistence within the nasal niche.

A critical consequence of these microbial shifts is the establishment of biofilm-associated communities, particularly in chronic or recurrent ENT conditions (74). Biofilm formation enhances bacterial survival by protecting microbes from host immune responses and antimicrobial treatments. In diseases such as otitis media with effusion, chronic rhinosinusitis, and adenoid hypertrophy, biofilms have been detected on mucosal surfaces and lymphoid tissues, serving as reservoirs for persistent inflammation and recurrent infection. Within these biofilms, microbial interactions differ substantially from planktonic states, favoring cooperative behaviors, metabolic cross-feeding, and resistance to environmental stress (74).

Importantly, pathogenic invasion initiates self-reinforcing feedback loops between inflammation, epithelial damage, and microbial restructuring. Inflammatory responses lead to epithelial disruption, impaired mucociliary clearance, and altered antimicrobial peptide production, further destabilizing the microbial ecosystem (75). This inflammatory milieu selectively favors inflammation-tolerant or pro-inflammatory taxa, perpetuating dysbiosis and preventing re-establishment of a commensal-dominated state. Over time, these feedback mechanisms may contribute to the transition from acute infection to chronic ENT pathology.

Collectively, these observations underscore that pediatric ENT diseases are not driven solely by the presence of individual pathogens but rather by ecosystem-level dysregulation of the nasal microbiome. Pathogenic invasion acts as a catalyst for microbial community collapse, functional reprogramming, and chronic inflammation. Understanding these dynamic microbial shifts is essential for identifying early biomarkers of disease progression and for designing therapeutic strategies aimed at restoring microbial equilibrium rather than indiscriminate pathogen eradication.

## 6 Knowledge gaps and methodological challenges

Throughout time, several research studies have brought significant information in understanding the nasal microbiome and its importance in pediatric ENT pathologies. Several gaps in knowledge and methodological limitations persists as obstacles to clinical implementation.

One of the major challenges in advancing nasal microbiome research in pediatrics lies in the methodological variability across studies. First, there is marked heterogeneity in sampling approaches. Studies differ in the anatomical site targeted (e.g., anterior nostrils vs. nasopharynx), swab technique and DNA extraction protocols, all of which impact microbial yield and comparability across cohorts (53). Sampling strategies, sequencing approaches, and analytical pipelines all strongly influence the reported microbial profiles, making it difficult to compare findings across cohorts or to derive reproducible conclusions (76–78). The site of sample collection is a central issue. While many studies rely on swabs from the anterior nares due to their non-invasiveness and ease of collection in children, these samples may not accurately reflect the microbial communities residing deeper in the nasopharynx, which are more relevant to otitis media and chronic rhinosinusitis. Nasopharyngeal aspirates and swabs, although more representative, can be uncomfortable and technically challenging, particularly in younger children (79). An additional layer of complexity arises from studies that analyze adenoid or tonsillar tissue collected during surgery. These samples may provide unique insight into biofilm-associated microbes and localized dysbiosis but are typically limited to children already undergoing surgical procedures, introducing selection bias. The lack of consensus on the optimal sampling site complicates the interpretation of results, as microbial composition varies across anatomical niches, and highlights the urgent need for standardized and clinically validated sampling protocols.

Current evidence comes from cross-sectional studies, which provide limited insight into the dynamics of microbial colonization through time and progression to diseases or health. Longitudinal studies are essential to establish causality and identify early microbial predictors of ENT pathologies (32, 43).

Functional potential of the nasal microbiota is still underexplored. While 16S rRNA sequencing reveals community structure, few studies assess gene expression (metatranscriptomics), metabolite production (metabolomics) or direct host-microbe interactions at the mucosal interface (5). Most current pediatric ENT microbiome studies rely on 16S rRNA sequencing, which, while useful for community-level taxonomic profiling, offers limited resolution and fails to capture functional potential. Shotgun metagenomics provides species- and strain-level resolution and allows the identification of antimicrobial resistance genes, but it is still underutilized in pediatric populations due to cost and sample size limitations. The next frontier is to combine genomic data with functional layers such as metatranscriptomics, which assesses microbial gene expression; metabolomics, which identifies host- and microbe-derived metabolites such as short-chain fatty acids, lipid mediators, or volatile organic compounds; and proteomics, which characterizes host–microbe interactions at the protein level (80–82). This integrative strategy could uncover mechanistic links between microbial communities and mucosal immune responses, offering a more comprehensive understanding of disease pathophysiology. For example, correlating the abundance of *Corynebacterium* and *Dolosigranulum* with specific anti-inflammatory metabolites or cytokine patterns could clarify their protective role in upper airway homeostasis. Such functional insights are critical for moving from descriptive associations toward actionable therapeutic targets.

Another critical gap is the lack of geographic and sociodemographic diversity. Most studies are based in North America and Western Europe, with limited representation of Eastern European or low- and middle-income pediatric populations (46). Local antibiotic use, pollution levels, allergen exposure and genetic background likely shape microbial profiles and disease risk but are rarely controlled for.

Many research studies published failed to corroborate clinical metadata such as symptom severity, treatment history, allergy status or comorbidities, essential variables for correlating microbial patterns with clinical expression.For reproductible findings and dependable translation into diagnostics and interventions, it is crucial to address the methodological limitations.

Finally, the field urgently requires greater standardization across study design and analytical pipelines. Currently, heterogeneity in DNA extraction kits, sequencing platforms, primer selection, and bioinformatics workflows leads to substantial variation in microbial profiles, even when analysing the same sample type. The absence of reference datasets specific to pediatric nasal microbiota further complicates benchmarking and interpretation. International collaborations and multi-centre studies could help establish harmonized protocols, including guidelines for sample collection, storage, sequencing, and data analysis. Equally important is the incorporation of robust clinical metadata—such as antibiotic use, vaccination status, environmental exposures, allergy history, and socioeconomic background—to allow meaningful correlations between microbiome patterns and clinical outcomes (83). Without standardized methodologies and comprehensive metadata, the translational potential of pediatric ENT microbiome research will remain limited. The establishment of shared databases and open-access repositories for pediatric nasal microbiome data would represent a crucial step toward reproducibility, comparability, and clinical application.

An important challenge of the current evidence base is the substantial methodological heterogeneity across studies investigating the pediatric nasal microbiome. Variability in sampling sites—including anterior nares, nasopharyngeal swabs or aspirates, and adenoid or tonsillar tissue—along with differences in laboratory and analytical workflows, limits cross-study comparability and complicates interpretation of reported microbial patterns.

In addition, the geographic distribution of available studies represents a further limitation. Most published data originate from Western Europe and North America, with limited representation from Eastern Europe and low- and middle-income regions. Given that environmental exposures, antibiotic practices, socioeconomic factors, and host genetics may influence nasal microbiome composition, this geographic imbalance restricts the generalizability of current findings. This underscores the need for standardized methodologies, harmonized analytical pipelines, and more geographically diverse, multi-center pediatric cohorts in future nasal microbiome research.

## 7 Future directions and clinical applications

Translational gap between microbiome research and clinical otorhinolaryngology must be reduced through not only methodological refinement but also a shift toward patient-centred applications.

A key direction is the development of microbiota-based risk stratification scores. Several studies suggest that certain microbial profiles – such as low abundance of *Dolosigranulum* or dominance of *Haemophilus* or *Staphylococcus* species – correlate with susceptibility to recurrent ENT infections and allergic inflammation in children (11, 44). These patterns could be used as predictive risk scores for early diagnosis or prognosis.

At the same time, there is growing interest in microbiome-informed diagnostics. Efficiently profiling could help in differentiating between viral, bacterial and dysbiotic causes of common otorhinolaryngological pathologies, particularly in situations which may require antibiotic treatment (53). The identification of microbial biomarkers may improve decision-making in borderline surgical cases such as adenoidectomy or tympanostomy tube placement.

Another promising frontier is therapeutic modulation of the nasal microbiome. While gut-focused interventions (e.g., probiotics or fecal microbiota transplant) are well-studied, nasal strategies are still emerging. Topical probiotics or bacterial-derived immunomodulators could offer non-invasive alternatives to systemic antibiotics or corticosteroids (19, 54).

In addition, future studies should adopt a multi-kingdom microbiome approach that integrates bacterial, viral, and fungal communities with AMR gene profiling. Understanding these complex interactions may help explain inter-individual variability in disease susceptibility and treatment response. Incorporating virome, mycobiome and resistome data into predictive models could lead to more precise risk stratification and innovative therapeutic interventions in pediatric ENT care.

Moreover, new evidence supports the possibility of personalized microbial interventions, guided by baseline microbial structure, environmental risk exposures (e.g., passive smoke, urban pollution) and immune phenotype. In the future, microbiota profiling may transform preventive care for high-risk children (e.g., those with recurrent upper airway infections or comorbid allergic rhinitis) (81).

To realize this potential, interdisciplinary collaboration is crucial – combining clinical otorhinolaryngology with microbiology, immunology, bioinformatics and public health. Large, multi-centre pediatric cohorts and controlled interventional trials will be essential to validate safety, efficacy and adaptability of microbiome-targeted therapies.

As research on the pediatric nasal microbiome advances, several future priorities emerge that could accelerate translation into clinical practice. One critical need is the establishment of large-scale, longitudinal cohorts that track microbial development from birth through adolescence. Such studies would provide essential insight into how early colonization patterns influence susceptibility to otitis media, allergic rhinitis, chronic rhinosinusitis, and obstructive sleep apnea, while clarifying causal relationships between dysbiosis and disease. Closely linked to this is the expansion of research into underrepresented populations, including children from Eastern Europe, Asia, and low- and middle-income countries. Broader geographic and ethnic representation will help identify population-specific microbial signatures, account for differences in environmental exposure, and ensure that microbiome-informed strategies are globally relevant (82).

Equally important is the adoption of multi-omics approaches that go beyond descriptive 16S rRNA studies. Integrating metagenomics, metatranscriptomics, metabolomics, and proteomics with host immunological profiling will generate a more functional understanding of microbial–host interactions at the mucosal surface (83). These data will also support the identification of microbial metabolites and immune-modulatory molecules that may serve as therapeutic candidates. To ensure reproducibility and comparability across studies, standardized sampling protocols, sequencing platforms, and bioinformatics pipelines must be developed and widely adopted, ideally through multi-centre collaborations and consensus guidelines.

Another major priority is the exploration of microbiome-targeted interventions through well-designed clinical trials. Topical probiotics, commensal-based nasal sprays, bacteriophage therapy, and immunomodulatory metabolites represent promising strategies, but their safety, efficacy, and long-term effects must be rigorously evaluated in pediatric populations. At the same time, advances in point-of-care sequencing and artificial intelligence should be harnessed to develop clinically applicable diagnostic tools capable of stratifying surgical risk, guiding antibiotic use, and predicting treatment response in real time.

Alongside diagnostics, microbiota-targeted therapies are emerging as a frontier in pediatric ENT. Experimental studies suggest that intranasal delivery of commensal organisms such as *Corynebacterium accolens* or *Dolosigranulum pigrum* could competitively exclude pathogens and promote immune tolerance (4). This strategy offers an attractive alternative to systemic antibiotics, with fewer collateral effects on microbiome diversity. Other promising avenues include bacteriophage therapy, where precision phage formulations targeting *S. aureus* or *H. influenzae* offer the possibility of eradicating pathogens while sparing beneficial taxa (84). Although still in preclinical stages for ENT use, phage therapy represents a paradigm shift in infectious disease management. Moreover, beneficial microbes produce bioactive molecules such as short-chain fatty acids and free fatty acids that strengthen mucosal barrier function and regulate immune responses. Harnessing or mimicking these metabolites could provide a new class of anti-inflammatory or barrier-protective therapeutics. In the future, combination strategies that integrate probiotics, phages, and immunomodulators may allow clinicians to re-establish microbial equilibrium while simultaneously reducing pathogen load and inflammation.

One of the most significant translational opportunities lies in the development of real-time microbiome analysis at the point of care. Advances in portable sequencing platforms, such as the Oxford Nanopore MinION, and rapid PCR-based microbial panels now enable same-day profiling of microbial communities (85, 86). In clinical practice, point-of-care sequencing could allow differentiation of viral infections from bacterial or dysbiotic states, thereby reducing inappropriate antibiotic prescriptions and supporting antimicrobial stewardship. Longitudinal sequencing performed during follow-up visits could also provide valuable information on microbial recovery after interventions such as adenoidectomy or tympanostomy, enabling more individualized treatment planning. The integration of microbiome data into electronic health records and its combination with artificial intelligence has the potential to generate predictive models that correlate microbial signatures with disease trajectories. Such AI-driven decision support tools could help clinicians identify children most likely to benefit from early interventions, making microbiome-informed care an integral part of precision medicine (87).

Perhaps the most transformative application of microbiome research lies in prevention. Indeed, the integration of microbiome data into preventive strategies holds the potential to transform pediatric ENT care. Identifying children at high risk for recurrent infections or allergic conditions based on microbial profiles, and tailoring preventive interventions accordingly, would represent a paradigm shift from reactive treatment to proactive health maintenance. The nasal microbiome may serve as an early-life biomarker for susceptibility to ENT disorders. Children with a history of recurrent otitis media, allergic rhinitis, frequent antibiotic exposure, or exposure to environmental risk factors such as passive smoke or urban pollution represent high-risk groups who might benefit from microbiome screening. Preventive strategies could then be tailored to individual risk profiles, ranging from early probiotic supplementation to targeted vaccination approaches guided by colonization patterns, as well as environmental modifications designed to reduce pollutant or allergen exposure. In the longer term, the combination of baseline microbial profiles with host genetic and immunological markers could allow the development of individualized prevention programs. This would mark a shift from reactive management of established disease to proactive maintenance of mucosal health.

## 8 Limitations

A limitation of the present review is its exclusive focus on pediatric populations. Early life represents a critical window for nasal microbiome establishment, during which host–microbiome interactions are particularly dynamic as a result of immune system immaturity, ongoing epithelial and mucosal development, and frequent environmental exposures. These features are associated with an increased susceptibility to conditions such as otitis media, adenoid hypertrophy, and allergic rhinitis, which are considerably more prevalent in childhood than in adulthood. However, restricting the analysis to this developmental stage limits the ability to directly extrapolate findings across the lifespan. Host–microbiome interactions undergo substantial age-related changes, including immune maturation, stabilization of epithelial barrier function, regression of lymphoid tissue hypertrophy, and the development of a more resilient and stable microbial ecosystem. These processes are thought to contribute to the reduced incidence and severity of many pediatric ENT conditions in adulthood. As a result, the microbial patterns and mechanistic pathways discussed in this review may not fully apply to adult populations. To avoid overgeneralization and to preserve mechanistic coherence, the scope of this review was therefore intentionally restricted to pediatric cohorts, where microbiome-driven effects are most pronounced and clinically relevant. Future studies adopting a life-course approach will be necessary to clarify how nasal microbiome–host interactions evolve from childhood into adulthood and how these changes influence ENT disease risk across different age groups.

Another important limitation of the current body of evidence, and consequently of this review, relates to the heterogeneity of study designs used to investigate the influence of seasonality, diet, and environmental exposures on the nasal microbiome. The available literature comprises a combination of longitudinal cohort studies and cross-sectional observational analyses, with variable methodological rigor and analytical depth. While some studies were specifically designed to assess seasonal variation or environmental exposures, many relied on indirect associations derived from temporal sampling or epidemiological correlations rather than controlled or hypothesis-driven experimental designs.

As a result, causal relationships between individual external factors—such as dietary patterns, seasonal changes, pollution, or household exposures—and nasal microbiome composition cannot be conclusively established. These limitations constrain the strength of inferences that can be drawn regarding the relative contribution of specific environmental determinants to microbial stability or dysbiosis.

A further limitation of the existing literature, and consequently of this review, is the predominant reliance on 16S rRNA gene sequencing in pediatric ENT microbiome studies. While this approach provides valuable information on community-level taxonomic composition, it offers limited resolution at the species or strain level and does not allow direct assessment of functional potential, metabolic pathways, or antimicrobial resistance determinants. Only a small proportion of studies have employed shotgun metagenomic sequencing, largely due to higher costs, low microbial biomass in pediatric nasal samples, and technical and ethical challenges associated with sampling in children. This methodological imbalance restricts the depth of mechanistic insight that can be derived from current data and underscores the need for broader adoption of high-resolution and multi-omics approaches in future pediatric nasal microbiome research.

## 9 Conclusions

In recent years, advances in sequencing technologies have deepened our understanding of the nasal microbiome’s role in pediatric health and disease. Far from being a passive colonizer, the nasal microbiota actively shapes local immune development, mucosal defense and susceptibility to chronic ENT conditions.

Several studies have demonstrated a distinct microbial pattern in children with otitis media, adenoid and tonsillar hypertrophy, allergic rhinitis and chronic rhinosinusitis. Dysbiosis – marked by reduced microbial diversity and dominance of potentially pathogenic genera – appears to contribute to disease persistence and recurrence. Presence of commensal taxa such as D. *pigrum* and C. *accolens* has been consistently associated with mucosal stability and reduced inflammation.

Clinical implementation of current knowledge is disrupted by methodological inconsistencies, cross-sectional study designs and the limited functional characterization of nasopharyngeal microbiota patterns. Longitudinal studies, standardized protocols and combined omics analysis approaches are needed to better determine causal relationships and therapeutic targets.

Looking ahead, the nasal microbiome holds promise as both diagnostic biomarker and a therapeutic target in pediatric otorhinolaryngology. Microbiota-based risk stratification personalizes microbial therapies and the prevention of dysbiosis-related disease may become part of routine clinical care – provided that the future research addresses the current gaps with rigor and translational intent.

As a narrative review, this work does not include formal quality assessment or risk of bias analysis of the included studies. Although the literature selection followed a defined strategy, the possibility of selection or publication bias cannot be entirely excluded.

This review highlights not only the importance of nasal microbiota in children and the complex relationship with local immune system but also its potential to revolutionize the prevention and treatment of common ENT disorders, bridging the gap between microbiology and clinical application which represents both a scientific and clinical frontier.
